# Use of spheroids as a model to evaluate the anticancer action of animal venoms and derived molecules: 2010-2024 review

**DOI:** 10.1590/1678-9199-JVATITD-2024-0068

**Published:** 2025-05-30

**Authors:** Yenny Yolanda Lozano Jiménez, Juan Daniel Hernández Vargas, David Mateo Navarrete Benavides, Ruth Mélida Sánchez Mora

**Affiliations:** 1 School of Basic and Applied Sciences, University of La Salle, Bogotá, Colombia.; 2 School of Health Sciences, University College of Cundinamarca, Bogotá, Colombia.

**Keywords:** 3D cell culture, Venoms, Antineoplastic agents, Complementary therapies

## Abstract

**Background:**

Cancer is one of the leading causes of death worldwide, with incidence rates continuously increasing, thereby posing a major healthcare challenge. Although many oncological drugs fulfill therapeutic requirements, they often show high toxicity due to their limited specificity. To address this problem, there has been a search for natural therapies, including animal venoms that harbor bioactive molecules with therapeutic potential, as well as biological models that facilitate their study. Consequently, three-dimensional culture models, such as spheroids, play a pivotal role in evaluating anticancer molecules, as they can effectively mimic *in vivo* tumor microenvironments.

**Methods:**

This study aimed to establish the significance of spheroids in identifying venom-derived molecules as potential therapeutic alternatives against cancer, based on a systematic review conducted from 2010 to 2024. Following PRISMA guidelines, a systematic search was conducted in four databases using the terms “Spheroid” and “Venom”. Of the 93 articles identified, 16 satisfied the inclusion criteria for this review.

**Results:**

Notably, several bioactive molecules derived from snake, spider, scorpion, and bee venoms were evaluated using various spheroid formation methods. These molecules demonstrated cytotoxic effects that impaired spheroid formation and disrupted invasion and migration processes.

**Conclusion:**

Overall, the findings indicate that the integration of three-dimensional culture models with venom-derived compounds constitutes a promising preclinical strategy for the development of innovative, venom-based therapeutic strategies for cancer treatment.

## Background

Cancer is a multifactorial disease influenced by both external factors such as exposure to chemicals, infectious organisms, and unhealthy diets, and internal factors, including inherited genetic mutations, hormonal imbalances, and immune conditions. These factors may act synergistically in enhancing the accumulation of one or more genetic or epigenetic events that trigger the onset of this disease [1, 2]. These events result in an uncommon acceleration and multiplication of a set of aberrant cells that lose the ability to undergo apoptosis, resulting in accumulations of cancer cells that can disseminate to other parts of the body [3, 4].

Globally, cancer is considered an important public health issue, being one of the main causes of morbidity and mortality [5, 6]. In 2022 alone, there were close to 20 million new cases and nearly 9.7 million deaths were recorded, corresponding to one in nine men and one in twelve women dying from it [7]. Over the past three decades, however, there has been a significant increase in the estimated 5-year relative survival for several types of cancer. This increase is partly due to development of oncology drugs, designed to meet rigorous therapeutic standards. Despite these advances, there remains a considerable risk of toxicity, attributable to low specificity of these treatments, which affects both healthy and cancerous cells [5, 6]. The choice of anticancer therapy is determined by multiple factors, including the type and stage of cancer, as well as the patient's general condition. Although treatment modalities include radiotherapy, surgery, immunotherapy, and hormonal therapies, chemotherapy remains predominant despite its side effects, such as cytopenia, nausea, vomiting, hair loss, and involvement of other organs and tissues [8, 9]. These adverse effects, often induced by reactive oxygen species and free radicals [10] can compromise treatment efficacy, sometimes requiring dose reductions or discontinuation [11]. Thus, there is a need to develop new therapeutic strategies that target more selective and less toxic active principles [12].

The search for effective cancer treatments has increasingly focused on the development of drugs derived from natural resources [13]. Bioactive compounds from animals, plants and bacteria have been used in the development of new drugs for diseases such as thrombosis, cancer, and human immunodeficiency virus (HIV), owing to their ability to induce angiogenesis, inhibit protein synthesis, trigger apoptosis, and exert antiviral effects, among others activities [14]. In particular, animal venoms have been of interest because they comprise a complex mixture of bioactive molecules with high affinity for multiple cellular targets. Their inherent toxicity also makes them valuable tools for investigating physiological and pharmacological processes that can guide drug development [15]. For instance, scorpion venom, has demonstrated the ability to inhibit the growth of various types of cancer cells via mechanisms such as ion channels blockade, binding to specific non-ion channels sites on the plasma membrane, and induction of apoptosis through activation of intracellular pathways [16]. Similarly, snake venom has been found to inhibit cancer cell proliferation by inducing apoptosis, modulating the expression of cell cycle regulatory proteins, and interacting with specific membrane sites [17]. Furthermore, bee venom contains key components such as melittin and PLA_2_, which present a synergistic cytotoxic effects on various cancer cell lines [18], by inducing apoptosis through caspase-dependent pathway or by creating transient or permanent pores in the phospholipid bilayer, leading to cell membrane rupture [18, 19]. It is important to note that investigating mechanisms of action of these therapeutics molecules requires the use of biological models that closely mimic physiological conditions, thereby providing essential information on the efficacy and safety of new treatments prior to clinical application.

### General overview of three-dimensional models in cancer research

Traditionally, two-dimensional (2D) culture systems, in which cells are grown as a monolayer on a flat solid surface, have been employed to search for new therapies. However, these systems suffer from limited cell-cell and cell-matrix interaction, which prevents them from replicating the complexity, heterogeneity and dynamic nature of human tumor microenvironments [20, 21]. Moreover, cells cultured in 2D often undergo cytoskeleton rearrangements that result in artificial polarity and aberrant gene and protein expression [21]. Consequently, three-dimensional (3D) culture methods, which incorporate components of the extracellular matrix (ECM), tumor cells, and stromal elements, are increasingly preferred as they foster extensive cell‑cell and cell‑matrix interactions and elicit responses more akin to *in vivo* conditions [22]. Indeed, 3D cultures exhibit differential gene expression, particularly in genes encoding signal transduction proteins and cell surface markers, thereby closely resembling *in vivo* tissues [23].

In general, 3D culture models are *in vitro* reconstructions of the ECM, preserving its geometric, mechanical, and biochemical properties [24]. These models consist of various cell types organized into structures that mimic natural tissues [24-26]. Their popularity has spread due to their capacity to maintain heterogeneity, cell topology, and cell-matrix interactions [27]. Consequently, 3D models enable detailed studies of cell morphology and organization shaped by ECM interaction, which are altered during oncogenic transformation. These models serve as indispensable tools for investigating cancer growth and metastasis mechanisms [24], offering more physiological environments relevant environments than 2D cultures while being cost‑effective and amenable to high‑throughput screening (Table 1) [28]. 

**Table 1.** Key characteristics of preclinical models used in cancer research.

Characteristic	Culture 2D	Spheroids	Organoids
Cost	Low	Low	Medium
Time	Low*	Low*	Medium*
Management	+++	++	+
Success radius	High	High	Medium
Yield potential	High	High	Medium
Heterogeneity	Without retention	Partial retention	Retention
Genetic manipulation	+++	+++	+++
Human immune components	-	+	+
Tumor-microenvironment interactions	-	++	++

The characteristics are scored as follows: low* (< 1 month), medium* (1-3 months), high* (several months), optimal (+++), good (++), adequate (+), not suitable (-). Adapted from Zanoni et al. [29].

On the other hand, the application of 3D cultures presents challenges including difficulties in stabilizing the cultures and the need for specialized materials. Despite these challenges, 3D cultures have emerged as excellent models for studying the biological mechanisms involved in cancer initiation and progression [27]. Among these models, organoids and spheroids stand out [28].

Organoids are structures that are formed in 3D cultures from stem cells isolated from primary patient samples. Their complexity is regulated by the cells inherent ability to self-organize and proliferate within a matrix, enabling the creation of biobanks representing diverse cancer types from multiple patients [30, 31]. Organoids are primarily used for epithelial-translation research, patient-specific treatment planning, and disease modeling due to their high fidelity to native tissue architecture and function. 

This model has provided compelling evidence of its accuracy in recapitulating the pathophysiological molecular, genetic, morphological, functional, and architectural features of cancer [31]. However, organoid technology still faces challenges, from the heterogeneous efficiency in deriving organoids from different tumor types and patients, as well as difficulties in integrating vasculature, stroma, and immune cells, although successful preliminary co-cultures have been described recently [30, 32]. One of the main limitations of organoids is the use of Matrigel (a reconstructed basement membrane extract secreted by a mouse sarcoma) for *in vitro* ECM modeling. Its animal origin leads to batch-to-batch variability, undefined composition and the presence of growth factors that may compromise the reproducibility of the model and introduce confounding factors [30]. 

Conversely, spheroids, like organoids, are self-organized 3D culture models, composed predominantly of tumor cells exhibiting a rounded morphology and extensive intercellular interactions. They are widely used due to their simple application protocols, high efficiency, and cost‑effective production [24, 33] (Table 1). Spheroids are indispensable for accurately replicating the tumor microenvironment, as they include both cancer cells and stromal components typically present *in vivo*, facilitated by mechanical dissociation of tumor tissues [33]. This arrangement, absent in 2D culture formats, better simulates *in vivo* drug delivery processes [34]. Moreover, the tightly packed three-dimensional structure of spheroids enables robust cell-cell interaction, including the formation of tight junctions analogous to those in native tissues, thereby establishing barriers for nutrient and drug transport. These properties make spheroids an improved platform for assessing drug delivery efficacy [34-36]. 

Furthermore, spheroids can incorporate normal cells within their microenvironment. To achieve this, co-cultures strategies have been developed to integrate multiple cell types into a single spheroid. This approach better replicates the *in vivo* intracellular signaling and tissue architecture, thereby enabling a more precise evaluation of the roles of diverse cellular components and their impact on drug delivery [34]. Co-cultures enable the integration of cancer stem cells while preserving their key properties, such as gene expression profiles, colony formation, and tumorigenic potential [34]. Additionally, these 3D models can develop central necrosis and hypoxic regions, features linked to drug resistance, making them essential for evaluating anticancer therapies and drug efficacy [34, 37]. 

These types of model also tend to form multicellular spheroids that, while sharing some conditions with 2D cultures, exhibit lower histological similarity to native tissue. Nevertheless, they retain metabolic and proliferative properties that demonstrate chemoresistance [33]. Models of this type provide advantages based on cell clonality, ease of maintenance, and genetic manipulation, making spheroids a suitable tool for high-throughput drug screening (Table 2). 

**Table 2.** Applications, advantages, and limitations of some methods used in spheroid generation.

Method	Applications	Advantages	Disadvantages	References
Hanging drop	Allows the study of tumor physiology, metabolism, cellular organization, and development. Suitable for co-culture and cell-cell interaction studies, making it effective for drug screening.	Simple to perform, spheroid size can be controlled by adjusting the number of cells. Spheroids are straightforward to scale and monitor.	Labor-intensive technique, challenging for large-scale production and long-term cultivation.	[38,39]
Liquid overlay	Facilitates investigation of tumor-fibroblast interactions and their role in tumor development.	Simple and easy to set up.	Achieving homogeneous spheroids is difficult, and there are limitations in mass transfer and cell viability.	[39]
ULA plates	Enables large-scale spheroid production.	Facilitates experimental reproducibility and monitoring of spheroid formation and growth. Inexpensive and easy to handle.	Some cell lines may not form tight spheroids.	[38]
External force techniques	Permits large-scale spheroid production.	Accelerates cell aggregation.	Requires specialized equipment and trained personnel; difficult to evaluate how external forces influence physiological cell changes.	[40]
Agitation-based techniques	Enables large-scale spheroid production and co-culture.	Provides dynamic, continuous culture conditions, supporting long-term cell viability.	Homogeneous spheroid formation is hindered. The approach is expensive and less effective in drug detection applications.	[39]
Microfluidic platforms	Applied in tissue engineering processes.	Offers design flexibility and reduced costs. Facilitates real-time cell monitoring and optimization of culture conditions.	Cell collection for analysis can be difficult.	[41,42]
Bioprinting	Used to develop complex structures through layer-by-layer approaches, allowing the modeling of living cells, biomacromolecules, and biomaterials to create 3D shapes from computer-aided designs.	Generates aggregates with uniform size and composition; allows co-cultures of different cell types. Can deposit live cells and growth factors simultaneously with biomaterials. Highly accurate and rapid.	Controlling the number/type of cells in individual droplets is challenging. High stress on cells, requires specialized equipment, and outcomes depend on the specific printing technique.	[38,39]
Spheroids encapsulated in a matrix	Suitable for analyzing cellular organization, modeling necrotic zones, assessing gene expression, and studying antibiotic resistance, among other applications.	Produces uniform spheroids without the need for sorting. The semipermeable membrane permits diffusion of nutrients, oxygen, small molecules, and debris.	Penetration of large macromolecules is limited. Not suitable for single-spheroid culture in HTS microwell plates; restricts spheroid size.	[38,43,44]

### Spheroids as three-dimensional models for tumor and tissue study

Spheroids are three‑dimensional cell aggregates that enable the study of both healthy and tumor tissues. They can be composed solely of cancer cells or include other cell types, such as fibroblasts, endothelial cells, or immune cells [43, 45]. The architecture of spheroids is designed to mimic the structure of natural human tumors. They contain an extracellular matrix composed of collagen, laminin, fibronectin, proteoglycans, and other components secreted by the constituent cells, which establish distinct cell‑cell and cell‑matrix interaction networks that differ markedly from those in monolayer cultures [43, 45]. Cells within spheroids grow in dense aggregates, which restricts the diffusion of glucose and oxygen, thereby simulating the conditions of a solid tumor [45]. Additionally, spheroids exhibit heterogeneous regions, with highly proliferative outer layers due to constant exposure to oxygen and nutrients. As these resources become limited, the proliferation rate declines, leading cells to enter a state of senescence and ultimately resulting in the formation of a necrotic core characterized by reduced pH due to the conversion of pyruvate to lactate [46]. Spheroid growth typically follows an exponential phase until reaching a diameter of 200-500 µm, after which growth plateaus to a stationary phase [46].

A variety of methods have been developed to generate spheroids of uniform size and consistency, including the hanging drop method, agitation‑based techniques, liquid overlay techniques, Ultra‑low attachment microplates, external force (acoustic, electrical, magnetic) methods, microfluidic platforms, and 3D bioprinting [47]. Table 2 summarizes the applications, advantages, and disadvantages of some popular spheroid generation techniques.

Given the advantages of spheroids as a cell culture model and the antiproliferative properties of animal venoms, a systematic review of the literature from 2010 to 2024 was conducted to establish the utility of spheroids in evaluating venom‑derived molecules as potential therapeutic alternatives for cancer.

## Methods

The study was conducted in accordance with the PRISMA (Preferred Reporting Items for Systematic Review and Meta-Analysis) guidelines established in 2009 [48]. An initial screening phase involved identifying records from electronic database followed by qualitative evaluation to select studies that provided valuable information on the animal‑derived molecules used to date, their effects, and their potential as therapeutic alternatives. 

All articles that addressed the therapeutic effects of animal‑derived molecules, particularly those investigating the impact of venom on cancer cell lines using spheroids as a 3D culture model, were considered eligible. Additionally, studies published between 2010 and 2024 in English and indexed in international medical databases that included information on the composition, bioavailability, and toxicological effects of these molecules were included. 

Articles published during this period were chosen due to the growing interest in these culture models. Selected articles underwent full‑text analysis for both qualitative and quantitative assessments. The inclusion and exclusion criteria were as follows:

- Original and review articles reporting on the effects of venom on cancer cell lines using spheroids as a cell culture model, including data on molecular composition, bioavailability, toxicological effects, and potential therapeutic applications.
- Studies that did not present relevant data in the medical field.

### Research and selection methodology

Articles were identified using the EBSCO, PubMed, Scopus and Web of Science electronic databases. Additionally, bibliographic references in the selected articles were reviewed to identify further potential studies. The search was performed using the terms “Spheroid” and “Venom” following the algorithm detailed in Table 3. 

To complement the search, a manual review of the articles included in the references was performed and relevant articles within the previously established search range were selected. Finally, screening was performed based on the title and abstract to eliminate records that were not related to the topic.

**Table 3:** Overview of the research methodology. Records identified in the databases: 93. Duplicates were removed using the tidyverse package in R-Studio software [49].

Database	Keywords	Search algorithm	Number of records	Records without duplicates
EBSCO	Spheroid Venom	(Spheroid* OR "3D culture" OR "3D model") AND Venom	10	
PubMed			24	
Scopus			38	
Web of Science			21	
Total records			93	45

## Results

**Figure 1.**
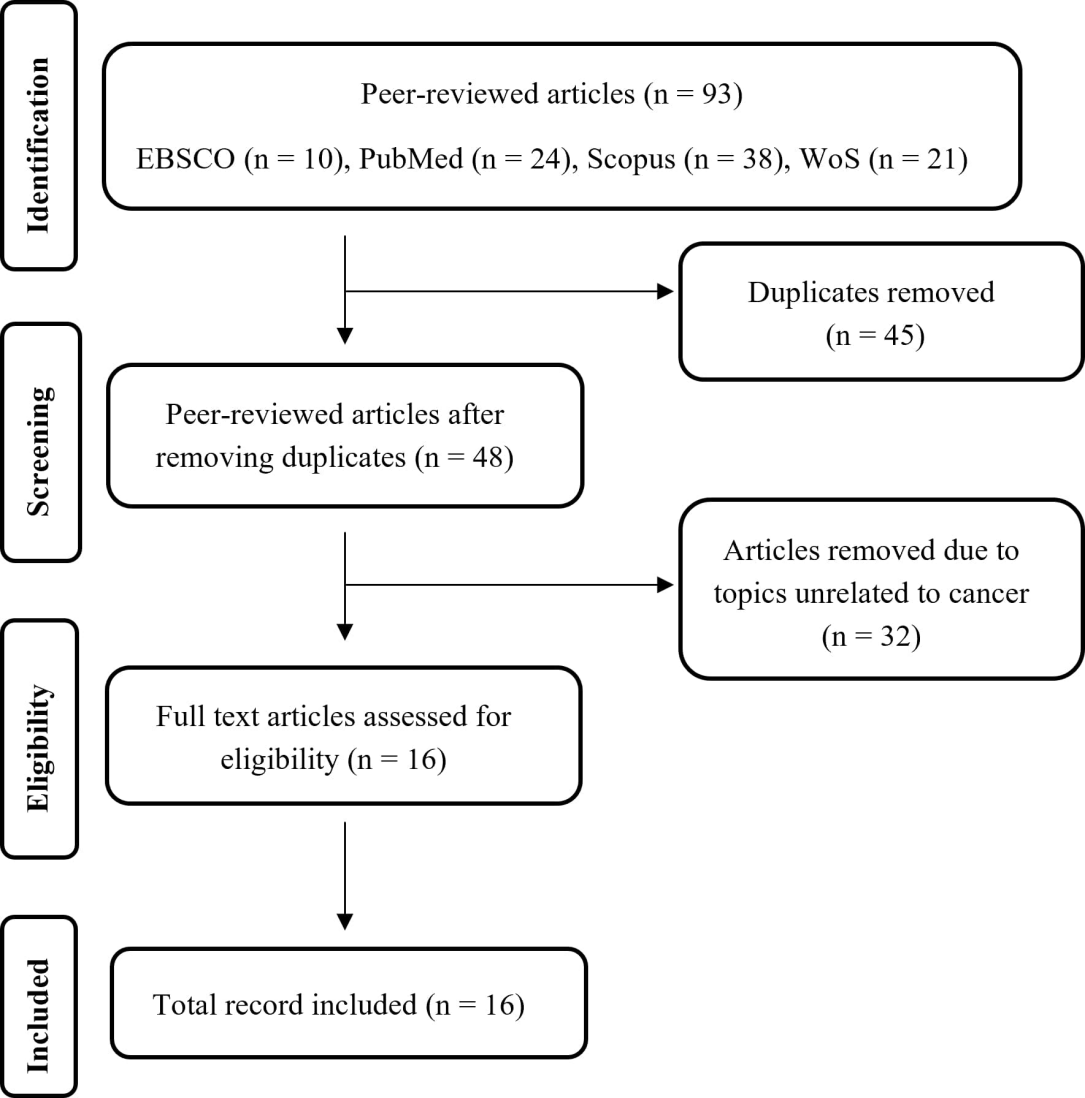
Flowchart outlining the studies that were included and excluded based on the PRISMA guidelines. WoS: Web of Science.

The database search (Scopus, PubMed, Web of Science, and EBSCO) identified 93 records. After duplicate removal using R‑Studio, 45 records remained. Following application of the inclusion and exclusion criteria, 16 articles were selected for the systematic review process. All selection and screening procedures are detailed in the flow diagram (Figure 1).

A total of 16 articles were obtained, 15 of which were primary research articles. Eight studies focused on molecules derived from snake venoms, with only one study employing whole venom [50]. Three studies investigated PLA_2_ [51-53], one examined the disintegrin accutin [54], another evaluated peptide [55] and two assessed metalloproteinases and L-amino acid oxidase [56, 57]. Additionally, three studies examined spider venom molecules, among which the use of peptides stood out, as they all employed the linear amphipathic α-helical peptide [58-60]. Furthermore, two articles employed molecules from scorpions, one using whole venom [61], and other evaluating venom-derived peptides [62]. Finally, two studies focused on molecules derived from bee venom, notably the cytolytic peptide melittin, with the latter studies incorporating drug delivery systems to enhance peptide functionality [63, 64]. 

In terms of culture methods, nine of the articles analyzed used ultra-low attachment plates [50-52, 54, 59-61, 63, 65], four used the hanging drop method [53, 55, 56, 62], one used the liquid overlay technique [57], and one utilized a combined method of liquid overlay and ultra-low attachment plates [64] (Table 4). One review by Kato’s group [66] highlighted the role of disintegrins as non-enzymatic substances detected in venoms derived from the snake families (Viperidae, Crotalidae, Atractaspididae, Elapidae and Colubridae), whose function is related to binding to certain integrins expressed by tumor cells and endothelial cells of the tumor microenvironment. They discussed the function of vicrostatin, a molecule synthesized from the disintegrin contortrostatin from the venom of the *Agkistrodon contortrix* snake, which was evaluated on spheroids of SKOV3 ovarian cancer cells. This study demonstrated that vicrostatin significantly inhibited tumor dissemination in SKOV3, reducing tumor growth by approximately 95-98% [66].

**Table 4.** Animal venom-derived molecules with anticancer potential evaluated in spheroids as a culture model.

Molecule/ venom (animal)	Cell line	Method	Cellular effects	Reference
Fractions from *Naja haje* venom (F4, F5, F7, F8)	LX2 Huh7.5 HUVEC WI38	Ultra-low attachment	Fraction F5 (10 µg/mL) reduced spheroid area. At 50 µg/mL, fractions F4, F5, F7, F8, and crude venom significantly decreased cell proliferation and spheroid size.	[50]
R-Lycosin-I (linear α-helical amphipathic peptide) from *Lycosa singoriensis* spider venom	A549	Ultra-low attachment	Exposure to the peptides induced physical contraction of the spheroids and a stronger reduction in cell viability compared to Lycosin-I. Both peptides penetrated the spheroids, with R-Lycosin-I reaching approximately 30 µm in depth, and Lycosin-I reaching around 10 µm	[60]
Crotoxin (CrTX), a PLA_2_ from *Crotalus durissus terrificus*	MRC-5 A549	Hanging drop	CrTX did not affect MRC-5 spheroid formation, but in co-culture with A549, it reduced spheroid size and decreased invasion by ~50%. It also inhibited MMP-9/MMP-13 secretion and cytokines/chemokines implicated in tumor progression, while downregulating mesenchymal markers (α-SMA, N-cadherin, αv integrin).	[53]
LVTX-9 and LVTX-9-C18 (linear α-helical peptides), from *Lycosa vittata* venom	B16-F10	Ultra-low attachment	At 20 µM, LVTX-9-C18 significantly reduced cell growth and viability, demonstrating higher cytotoxicity than unmodified LVTX-9.	[65]
*Buthus occitanus* scorpion venom, fractionated (F1-F7)	LX2 Huh7.5 HUVEC WI38	Ultra-low attachment	Fraction F3 (10 µg/mL) reduced spheroid cell area by 21.09%. The other fractions did not exhibit significant differences compared to the PBS control.	[61]
Synthetic crotamine analog (sCrot-Cy3)	SKMEL-28 B16-F10	Hanging drop	The peptide showed rapid internalization and a homogeneous distribution within the melanospheres, indicating robust uptake and uniform diffusion in tumor cells.	[55]
BthTX-II (Asp-49 PLA_2_) from *Bothrops*	MDA-MB-231 MCF10A	Ultra-low attachment	BthTX-II inhibited cell adhesion (57% with Matrigel, ~53% with fibronectin or collagen) and reduced migration (up to 60%) and invasiveness (up to 92%). It also suppressed key integrins and prevented spheroid formation in MDA-MB-231 cells.	[52]
P-I metalloproteinase (MP-1) and L-amino acid oxidase (LAAO) from *Bothrops moojeni* and *Bothrops atrox*	HUVEC	Hanging drop	MP-1 at 2 µg/mL inhibited angiogenic sprout formation, while LAAO significantly reduced sprout formation at 10 and 100 ng/mL, indicating anti-angiogenic potential.	[56]
BthTx-II (PLA_2_) from *Bothrops jararacussu* venom	MDA-MB-231 HUVEC	Ultra-low attachment	At 10 µg/mL, BthTx-II inhibited cell aggregation (particularly in endothelial cells). At 50 µg/mL, it reduced invasion, migration, and proliferation in co-cultures over 24 hours, with notable morphological changes in the HUVEC-MDA-MB-231 interaction.	[51]
CTX, CA4, CTX-23 (chlorotoxin derivatives) from *Leiurus quinquestriatus* and *Buthus martensii* scorpion venom	U251	The hanging drop	At 10 µM, these peptides significantly inhibited cell migration and reduced invasion area by approximately 20%, indicating antiglioma potential in 3D spheroids.	[62]
Melittin was modified with the photosensitizer chlorin Ce6 (MEL/Ce6) and used with or without hyaluronic acid (HA) coating, including FITC-MEL/Ce6 and FITC-MEL/Ce6@HA	A549	Ultra-low attachment	FITC and Ce6 were mainly at the spheroid periphery. After 670 nm irradiation, intense fluorescence signals of both were observed inside spheroids, even at 105 µm depth	[63]
Melittin in polyionic complex (PIC) micelles conjugated with estrone	MCF-7	Liquid overlay method and ultra-low attachment	Both free and micelle-incorporated melittin caused morphological changes and cell detachment in MCF-7 spheroids; however, PIC micelles reduced viability more effectively than free melittin, and estrone conjugation further enhanced cell death. In MDA-MB-231 spheroids, changes were milder, but nuclear internalization of the polymer was observed after 10 hours of treatment.	[64]
Lycosin-I and Lycosin-C12 (linear α-helical peptides) from *Lycosa singoriensis* spider venom	A549	Ultra-low attachment	Lycosin-C12 (5 µM) strongly suppressed spheroid growth; at 10 µM, it induced spheroid shrinkage and peripheral collapse. It decreased viability more effectively than Lycosin-I and inhibited spheroid migration at 2.5-10 µM without detectable cytotoxicity at 20 µM.	[59]
Pollonein-LAAO from snake *Bothrox moojeni* venom	PC-3 HFF-1	Liquid overlay	Pollonein-LAAO reduced PC-3 cell viability by approximately 40% at concentrations of 3.125-50 μg/mL, modulated the expression of pro-apoptotic and cell cycle arrest genes, and decreased spheroid area.	[57]
Accutin peptide from *Agkistrodon acutus* venom	A549 H1299, H460	Hanging drop.	Accutin induced dose-dependent effects in A549 cells, with 28 μM causing cell rounding and 0.922 μM inhibiting migration without affecting viability. Significant anti-migration activity was also observed at 0.922 nM in both A549 and H1299 cells.	[54]

## Discussion

Animal venoms have been employed by mankind due to their healing and medicinal properties since the beginning of civilization. Their therapeutic potential is attributed to their high selectivity and potency, as they contain neurotoxins, myotoxins, enzymes, and other bioactive substances. These characteristics have drive research into identifying venom components with pharmacologically active properties for the novel therapeutic development. However, the use of venom components as therapeutic agents has seen only moderate success [67], primarily due to the reliance on 2D culture models in preclinical cancer drug discovery, which fail to predict *in vivo* efficacy. This shortcoming contributes to a low success rate and increased cost in the clinical approval of new investigational drugs [44]. Recently, 3D models such as spheroids have gained recognition as an intermediate step between *in vitro* models and *in vivo* models, offering enhanced relevance in research fields such as tumor biology and drug screening [68], by enabling the assessment of tumor response and sensitivity to chemotherapeutic drugs, targeted therapy and drug delivery systems. Spheroids also facilitate high-throughput screening for both negative and positive drug candidate evaluations, thereby reducing the need for animal testing and supporting new drug development [41]. Consequently, spheroids are emerging as to be a fundamental tool for identifying alternative cancer therapies derived from animal venom. 

Method selection for tumor spheroid generation critically influences the interpretation of venom‐induced cytotoxicity and antitumor effects, as demonstrated by reviewed studies. The hanging drop method, as employed by Kato and Sampaio [53] and Mambelli-Lisboa et al. [55], is well‐suited for assessing complex cellular interactions in co-culture systems. For example, it facilitated the evaluation of CrTX-mediated invasion inhibition in A549/MRC-5 cells and ensured the homogeneous distribution of sCrot-Cy3 in melanospheres [53, 55]. Its main advantages include procedural simplicity and precise control over spheroid size, facilitating detailed analyses of cellular dynamics. However, this method exhibits limited scalability and reduced culture stability and uniformity, which explains the preference for the ultra-low attachment (ULA) method in high‐throughput studies. 

The ULA technique, as used by Zhang et al. [60] and de Vasconcelos Azevedo et al. [52], allows standardized spheroid production and accurate quantification of effects, such as spheroid area reduction (F5 fraction of *Naja haje* venom) or BthTX-II-induced inhibition of cellular adhesion [52, 60]. Nonetheless, difficulties in forming compact spheroids in certain cell lines, as observed with *Lycosa vittata* peptides [65], highlight the need of protocol optimization tailored to specific cellular models.

In contrast, although the liquid overlay method yields lower reproducibility due to spheroid heterogeneity, it remains valuable for studies emphasizing simplicity and tumor-stroma interaction analyses. For instance, Polloni et al. [57] used this technique to evaluate Pollonein-LAAO effects on PC-3 spheroids, reporting a 40% reduction in viability and increase of proapoptotic gene expression [57]. However, inherent limitations such as heterogeneity, restricted diffusion, and complex handling, may complicate the interpretations of compound penetration and viability. In contrast, the ULA method, by ensuring spheroid homogeneity and enabling continuous monitoring, was crucial for demonstrating deep penetration of peptides such as R-Lycosin-I (30 µm) [60] and the nuclear internalization of melittin-loaded micelles, findings essential for validating therapeutic strategies [64].

Collectively, these findings indicate that while the hanging drop and liquid overlay methods are optimal for mechanistic studies in simplified models, the ULA method offers greater robustness for large-scale screenings and pharmacokinetic analyses, provided the inherent limitations of each cellular system are considered. Thus, method selection should align with experimental objectives, prioritizing scalability, reproducibility, and biological relevance within the tumor microenvironment.

Furthermore, co-culture models dominate the reviewed studies because cell-to-cell interactions and the secretions of soluble factors within spheroids enhance tumor growth and progression by remodeling the protein composition of the ECM, inducing cancer cell migration, and promoting cancer invasion [69]. Specifically, co-culture with HUVEC cells is prominent, as it enables the formation of microvascularized tumor environments that allow the simulation of angiogenesis and facilitate the interaction between venom‐derived molecules, tumor cells, and stromal cells [69]. This is exemplified by Bhat et al. [56], who demonstrated that PI metalloproteinases and L-amino acid oxidase (LAAO), at concentrations of 2 µg/mL, modulate angiogenesis in the co-culture model [56]. While spheroids derived from HUVEC cells effectively replicate vascular architecture and function, which is crucial for studying angiogenesis in response to isolated compounds, fractions, or crude venoms [56, 61] their use presents certain challenges. Specifically, the incorporation of additional factors, such as type I collagen, is necessary to ensure the formation of stable spheroids [70]. Furthermore, a meticulous experimental design is required to strictly control spheroid size and uniformity, thereby reliably simulating angiogenic processes [71]. 

The use of spheroids has significantly advanced preclinical research by providing an ideal platform to evaluate local penetration, cellular distribution, and binding properties of various molecules [72]. In this model, candidate molecules must penetrate the three-dimensional spheroid structure to demonstrate their efficacy, enabling a more precise assessment of their bioavailability and mechanism of action. This review emphasizes studies focused on peptides, which typically utilize optical tools to monitor this process. Notably, Jia et al. [63], used confocal microscopy to reveal the presence of the modified melittin peptide, MEL/Ce6@HA, on the membrane of A549 cancer cells [63]. Similarly, fluorescence microscopy studies on the peptides sCrot-Cy3 and R-Lycosin-I demonstrated their high penetration capacity, with broad and uniform distribution within spheroid [55, 60].

These findings indicate that animal-derived peptides exhibit remarkable specificity in their biological specificity, positioning them as promising candidates for the development of novel therapeutic strategies. However, their clinical application faces significant challenges, particularly in the evaluation of their efficacy and therapeutic safety [73]. Among the main obstacles is their high toxicity, which may induce adverse effects, and rapid proteolytic degradation, which substantially reduces bioavailability. These limitations compromise the peptides stability in the bloodstream and hinder effective target delivery.

The antitumor potential of animal venoms arises from the remarkable diversity of their bioactive components, including PLA_2_, matrix metalloproteinases (MMPs), L-amino acid oxidases (LAAOs), and cytotoxic peptides. These molecules play a crucial role in modulating key cellular processes, such as apoptosis induction, inhibition of angiogenesis, and remodeling of the tumor microenvironment, making them promising candidates for cancer therapy [8].

Among these bioactive compounds, PLA_2_ enzymes are notable for their hydrolytic activity on phospholipid membranes, which releases lysophospholipids and free fatty acids. This activity yields significant pharmacological effects, including membrane destabilization, degradation of membrane-bound proteins, and disruptions in intracellular signaling pathways. Collectively, these mechanisms inhibit tumor cell proliferation and angiogenesis, highlighting their potential as therapeutic agents [67]. In this context, *Naja haje* cobra venom has demonstrated a remarkable anticancer effect, particularly against hepatocellular carcinoma cells. The studies revealed that exposure to *Naja haje* venom significantly reduces spheroid size, which correlates with a decreased intensity of the RPD signal, an indicator of cell proliferation [50]. 

On the other hand, this review emphasizes the role of BthTx-II, PLA_2_ from the venom of the *Bothrops jararacussu* species, which significantly inhibited invasive effects in co‐cultures of triple‐negative breast cancer and endothelial cells, accounting for over 60% [51]. Additionally, this phospholipase, in 3D models, has the capacity to inhibit key factors during cell adhesion, migration and dissemination, such as integrin genes (α2, ß1, αvß3); with ß1 integrin involved in migration and αvß3 in angiogenesis - thereby offering an antitumor and anti‐metastatic strategy by inhibiting adhesion on matrices like collagen and fibronectin [52]. Similarly, crotoxin, a PLA_2_ from *Crotalus durissus* terrificus venom, modulates tumor cell adhesion in 3D cultures by reducing invasion area through inhibition of N‐cadherin, α‐SMA, and αv integrin expression in MRC‐5/A549 spheroid co‐cultures [53]. This suggests that crotoxin interferes with integrin-dependent actin polymerization, thereby impairing tumor cells migration [53]. Furthermore, crotoxin remodeling of the tumor microenvironment by regulating TGF‐β1 activation, via inhibition of MMP‐9 and αv integrin secretion in MRC‐5/A549 spheroids and by inhibiting MMP‐13 secretion, which activates MMP‐9; both actions are associated with reduced metastasis and invasion [53]. Finally, the presence of crotoxin in MRC-5/A549 spheroids markedly inhibits the binding of chemokines to their receptors, processes linked to proliferation, migration, invasion, and epithelial-mesenchymal transition in various cancer cell lines [53]. 

Snake venom-derived metalloproteinases (SVMPs) are major components of the venom of the Crotalidae and Viperidae families, which cause coagulation factor activation, inhibition of platelet aggregation, as well as hemorrhagic and fibrinolytic activities; thus, the anticancer activity of these metalloproteinases is implicated in proinflammatory and apoptotic effects [67]. Purified LAAOs have been shown to induce cell death by generating intracellular reactive oxygen species (ROS), leading to significant oxidative stress in tumor cells [57, 67]. For instance, Pollonein‐LAAO from *Bothrops moojeni* induces ROS accumulation that correlates with upregulation of genes such as TP53, a tumor suppressor that transcriptionally regulates apoptosis via pro‐apoptotic gene expression [57]. Both SVMPs and LAAOs isolated from *Bothrops atrox* and *Bothrops moojeni* have demonstrated the capacity to induce endothelial cell stress and inhibit capillary growth in 3D cultures. These findings imply cytotoxic effects on endothelial cells that may impede tissue regeneration and delay wound healing, offering potential strategies for anti‐angiogenic therapy [56]. 

Another notable snake venom component is crotamine, a major toxin in *Crotalus durissus* terrificus venom responsible for myonecrosis in envenomation. Crotamine shares high homology with myotoxins and resembles β‐defensins, which are cell‐penetrating peptides with specificity in modulating cell proliferation. It penetrates cell membranes to access intracellular targets, either in the cytoplasm or nucleus, thereby broadening its potential in preclinical applications [55, 74]. Studies demonstrate that crotamine effectively penetrates melanospheres, offering a valuable model for investigating stromal cell interactions within the tumor microenvironment, crucial for testing emerging therapies [55]. Additionally, accutin, a short chain disintegrin from *Agkistrodon acutus* venom, has been identified as a potent inhibitor of platelet aggregation and angiogenesis [75]. Its RGD motif stably binds to integrin α5β1, inhibiting migration and invasion in lung cancer cell lines at 9.22 nM [54]. 

In addition, arachnid venoms have demonstrated significant impacts on cancer‐related characteristics [15]. Scorpion venom and its derivatives serve as valuable tools in cancer treatment. They can alter membrane permeability or selectively bind receptor domains to induce cell death or inhibit growth via diverse signaling pathways. Additionally, these substances modify the tumor microenvironment, rendering it less conducive to cell survival, notably by inhibiting angiogenesis [12]. Recently, spheroid models have been instrumental in identifying novel molecules derived from chlorotoxins of *Leiurus quinquestriatus* and *Buthus martensii* scorpions. Chlorotoxins have been shown to reduce glioma cell growth, inhibit migration, and diminish tube formation by human endothelial cells, thereby curbing tumor‐induced angiogenesis [62]. Although many bioactive compounds from scorpion venom exhibit positive effects, some assays with *Buthus occitanus* venom in multicellular spheroids did not yield statistically significant results, except for one fraction, suggesting it contains potent bioactive compounds [61]. Thus, the evaluated fraction likely contains the peptide RK1, which exhibited inhibitory activity on proliferation and migration in U87 glioblastoma cells in 2D cultures [76]. The observed low cytotoxicity in spheroids may be attributed to their complex composition and organization, highlighting the importance of studying drug diffusion mechanisms to understand toxin transport within the tumor microenvironment [38, 63].

While less extensively studied than scorpion venom, spider venom also has therapeutic potential. Its primary components are small peptides with stable disulfide bridges, conferring resistance to proteolytic degradation and high specificity for key molecular targets [15]. These peptides represent a novel class of anticancer agents capable of targeting cells with high specificity and reduced toxicity in healthy tissues. For example, linear amphipathic α‐helical peptides have demonstrated inhibitory effects on tumor cell growth *in vitro* [15]. Notably, Lycosin‐I a peptide from *Lycosa singoriensis* venom when applied to A549 lung cancer spheroids, induced physical contraction of spheroids and exhibited potent inhibitory effects due to its cytotoxicity, enhanced serum stability, and improved spheroid penetration [59, 60]. A similar linear amphipathic α‐helical peptide has been identified in *Lycosa vittata*. In this case, the peptide LVTX‐9 exhibited potent cytotoxic activity in tumor spheroid assays, underscoring its potential as a lead compound in anticancer drug development [58].

Finally, bee venom warrants attention due to its traditional use in oriental medicine for treating conditions such as arthritis, rheumatism, tumors, and skin diseases [77]. Recent studies report that bee venom induces apoptosis, necrosis, and cytotoxic effects, thereby inhibiting the growth of various cancer cell types [77]. These effects are attributed to its diverse active compounds, notably melittin, which constitutes 40-50% of the venom's weight [2, 77]. Melittin notably activates PLA_2_‐dependent pathways, critical for anticancer activity. Furthermore, conjugation of melittin with hormone receptors and gene therapy vectors may offer novel cancer‐targeting strategies [77]. These results underscore the importance of delivery mechanisms for melittin, as its high hemolytic activity and nonspecific cytotoxicity limit its direct use [63]. These mechanisms have been evaluated in spheroids of the A549, where the efficacy of melittin in producing membrane lysis was observed, greatly increasing the depth of tumor penetration in spheroid [63]. Moreover, the effects of free melittin and melittin incorporated into delivery systems have been assessed in MCF‐7cell. These experiments revealed significant morphological alterations, characterized by cell detachment from tumor spheroids, particularly with integrated delivery systems (Polyionic complex micelle and FITC‐MEL‐Ce6@HA), which protect melittin activity and sustain its effects on cell growth [64].

Although animal‐derived molecules remain largely experimental as cancer therapeutics, interest has surged with the advent of 3D models. Spheroids, in particular, offer a robust simulation of the tumor microenvironment, facilitating the identification and mechanistic study of anticancer compounds from snake and arthropod venoms. 

## Conclusions

Utilizing biological models that simulate the tumor microenvironment, including interactions among cancer cells, the extracellular matrix, and stromal cells via co-cultures, facilitates a more efficient exploration of alternative anticancer therapies that effectively and safely combat the disease. In this context, spheroids as a cell culture model constitute an excellent option for evaluating molecules derived from animal venoms, as they elucidate the anticancer activity of bioactive compounds and their mechanisms of action on each component of the tumor microenvironment.

This culture model has been used to investigate the therapeutic potential of snake venom molecules, including PLA_2_, L-amino acid oxidases, disintegrins, metalloproteinases, and toxins such as crotamine. Similarly, arthropod venom-derived molecules with potential anticancer activity were examined, including a linear amphipathic α-helical peptide from spiders, the cytolytic peptide melittin from bees, and chlorotoxins from scorpions, which demonstrated antiproliferative effects on spheroids and influenced protumoral mechanisms, such as invasion, migration, cell adhesion, and mesenchymal to epithelial transition, among others.

Consequently, investigating this culture model with natural substances, such as venom derivatives, represents a promising preclinical strategy. This approach not only elucidates the mechanisms of action of these substances but also facilitates the examination of their interactions with the tumor microenvironment, which is essential for anticancer drug development.

### Abbreviations

2D culture: two-dimensional cell culture; 3D bioprinting: three-dimensional bioprinting; 3D culture: three-dimensional cell culture; A549: a cell line derived from a human adenocarcinoma of the lung; B16-F10: a murine melanoma cell line that is highly metastatic; BthTX-II: *Bothrops asper* toxin II; CrTX: crotoxin; CTX: chlorotoxins; CTX, CA4, and CTX-23: three peptides derived from chlorotoxins; DEP: dielectrophoresis; DMEM: Dulbecco's modified Eagle medium; ECM: extracellular matrix; FBS: fetal bovine serum; FITC: fluorescein isothiocyanate; Fmoc: fluorenylmethoxycarbonyl; HBM: human brain medium; HTS microwell: high-throughput screening microplate wells; HFF-1: human foreskin fibroblasts, a cell line derived from neonatal foreskin tissue; H460: a human large cell lung carcinoma cell line; H1299: a human non-small cell lung carcinoma cell line with a non-functional p53 gene; HUVEC: human umbilical vein endothelial cells; HA: hyaluronic acid; LX-2: a human hepatic stellate cell line derived from activated hepatic stellate cells; LAAO: L-amino acid oxidase; LVTX-9: synthetic linear amphipathic α-helical peptide derived from the cDNA library of *Lycosa vittata*; MDA-MB-231: a cell line derived from a human triple-negative breast cancer; MCF-7: a human breast cancer cell line derived from ductal carcinoma; MCF10A: a non-tumorigenic human mammary epithelial cell line; MRC-5: a human diploid fibroblast cell line derived from fetal lung tissue; PC-3: a human prostate cancer cell line derived from an adenocarcinoma; PBS: phosphate-buffered saline; PLA_2_: phospholipase A_2_; PRISMA: Preferred Reporting Items for Systematic Review and Meta-Analysis; PRM1 Medium: Roswell Park Memorial Institute Medium; RP-HPLC: reverse-phase high-performance liquid chromatography; SKMEL-28: a human melanoma cell line; SKOV3: serous adenocarcinoma of the ovary 3; sCrot-Cy3: synthetic crotamine conjugated with a fluorescent dye Cy3; ULP plates: ultra-low attachment plates; WI-38: a human diploid fibroblast cell line derived from lung tissue; α-SMA: alpha-smooth muscle actin.

## Data Availability

Not applicable.
